# Loss in the Intrinsic Quality and the Antioxidant Activity of Sunflower (*Helianthus annuus* L.) Oil during an Industrial Refining Process

**DOI:** 10.3390/molecules27030916

**Published:** 2022-01-28

**Authors:** Larbi Rhazi, Flore Depeint, Alicia Ayerdi Gotor

**Affiliations:** 1 Institut Polytechnique UniLaSalle, Université d’Artois, ULR 7519, 19 Rue Pierre Waguet, BP 30313, 60026 Beauvais, France; Flore.Depeint@unilasalle.fr; 2 Institut Polytechnique UniLaSalle, AGHYLE, UP 2018.C101, 19 Rue Pierre Waguet, BP 30313, 60026 Beauvais, France; alicia.ayerdi-gotor@unilasalle.fr

**Keywords:** antioxidants, industrial refining, minor components, sunflower oil

## Abstract

Minor compounds in vegetable oils are of health interest due to their powerful biological antioxidant properties. In order to extend the shelf life of sunflower oil, it is generally subjected to a refining process that can affect these desirable compounds. The main purpose of this study was to determine the effect of this chemical/physical refining process on selected minor components of sunflower oil in order to establish the nutritional quality and health properties of the oil. The oxidative stability, contents of fatty acids, tocopherols, phytosterols, reducing capacity, β-carotene, chlorophyll, and squalene were studied during six refining steps. Quantitative data showed the evolution of oil quality according to its degree of refinement. The results showed a significant decrease for all of the minor compounds analyzed, with losses in carotenoids of 98.6%, 8.5% in tocopherols, 19.5% in phytosterols and 45.0% in squalene. The highest reductions were recorded for the compounds that alter the most the visual aspects of the oil (waxes, carotenoids and chlorophylls) whereas reduction was limited for the compounds with no impact on the organoleptic quality. The losses in the compounds of health interest should be minimized by improving the refining processes and/or having a greater content of those molecules in crude oil by breeding new performing varieties.

## 1. Introduction

Sunflower-oil extraction and the subsequent refining process is an essential step to maximize the production and preserve oil stability over time. Sunflower oil was the first oil consumed in Europe, and represented 17.6 g/capita/day or 35.6% of the total oil consumption in 2018 [1]. Sunflower oil is the fourth most consumed oil in the world, representing 4.8% in 2017-18, after palm, soybean and rapeseed oils with 47.2, 37.6 and 11.3%, respectively [1].

Oils have an important role in nutrition by providing energy and essential fatty acids (FA) [2]. Based on differences in oleic acid content, sunflower oil can be classified as (1) linoleic (14–39%), (2) mid-oleic (43–72%), or (3) high-oleic (HOSO) (75–91%) [3]. The percentage of polyunsaturated FA is generally higher than 90%. The conventional sunflower genotypes produce an edible oil with a high level of linoleic acid whereas the high-oleic sunflower genotypes produce an oil containing more than 80% of oleic acid [3,4]. FA composition is controlled by the genotype and the environmental conditions during the grain-filling stage [4,5,6]. Thanks to its good oxidation stability and its FA composition presenting a high ratio of polyunsaturated to saturated FA, linoleic sunflower oil has many applications in the food market, mainly as premium salad oil, as cooking oil or in oil mixtures. In industrial uses, its high thermo-oxidative stability and composition make sunflower oil a healthy option as a frying oil, with a preferential use of HOSO because of its low content of polyunsaturated FA [7]. Mid-oleic sunflower oil is the second-best option for frying, and is better than other oils (soybean, canola, corn and cotton) because of its composition, but its production in Europe is limited [8]. Over the last decade, sunflower oil has replaced palm and soybean oils in most of the potato chips and French fries sold in Europe [9]. It is also widely used in the manufacture of mayonnaise and oil-based seasonings. Hydrogenated sunflower oil can be used in the manufacture of shortenings and margarines. Besides, HOSO offers a lower oxidation rate and a higher temperature stability than traditional sunflower oil. Additionally, its triglycerides have good properties at low temperatures, which makes this oil interesting for non-food industrial uses such as biolubricants [10] and biodiesels [11]. 

It is undeniable that taste, nutritional quality and stability are the main factors determining the production, acceptance and marketing of vegetable-oil products [12]. These factors are determined by the intrinsic composition of a vegetable oil. They are particularly influenced by the nature and content of certain compounds including minor compounds such as free fatty acids, phospholipids, trace metals, waxes, tocopherols, phytosterols and polyphenols, which have pro- or antioxidant properties. It is therefore necessary to preserve their nutritional constituents in order to prevent oxidation and eliminate undesirable elements. The presence of minor components such as tocopherols, phytosterols or mineral elements is an important factor as they have an impact on the population’s health [13,14], but they can also increase the oil stability [15]. Besides, an improved oil shelf life is associated with the removal of undesirable elements such as heavy metals [16] or free radicals, which initiate the oxidation reaction [17]. The oil-refining process aims to produce edible oil while retaining the interesting minor components and eliminating minor substances that may modify or reduce the quality and the stability of the oil, such as phospholipids [18], pigments [19], peroxides [20], metallic ions [21] and volatiles [22]. Removing or reducing the content of these undesirable molecules will increase the shelf life of the oil but may also reduce those minor components that have a nutritional or technological interest.

There have been many studies investigating the losses in minor components, but they concentrated mainly on a part of the process [18,23,24] or on just few components of the entire extraction and refining process [25,26]. The review conducted in 2016 [27] showed that there were no studies on the evolution of all of the minor components during an industrial process. The objectives of this work are to evaluate the global losses in the most important minor components at each stage of the refining process of solvent extraction with a classic Linoleic oil (LSO) in order to identify and qualify each step and to evaluate the potential of using the residues to recover interesting by-products. 

## 2. Results

The purpose of refining is to maintain and/or to improve the organoleptic characteristics such as taste, neutral odor, limpidity or light-yellow color, and the stability of fats. To do this, several steps are implemented to remove undesirable compounds (gums, waxes, free fatty acids, pigments, metallic traces, volatile odorous compounds) and contaminants potentially present in the raw materials, while preventing the formation of new undesirable compounds by hydrolysis, oxidation or isomerization during ulterior uses of the oil such as frying [28]. The process control can, if necessary, be adapted to future uses of the refined oils produced: as food (or cosmetics/pharmaceuticals), which requires the refiner to optimize the process in order to preserve the constituents of nutritional interest (polyunsaturated fatty acids, vitamins E, etc.), while as agrofuel there is no need to preserve those constituents, but they can be valorized as by-products. In this study, a number of constituents were tracked. Additionally, the oxidative stability of the oil was also studied at different phases of the refining process. 

### 2.1. Cold Precipitates

**Figure 1:**
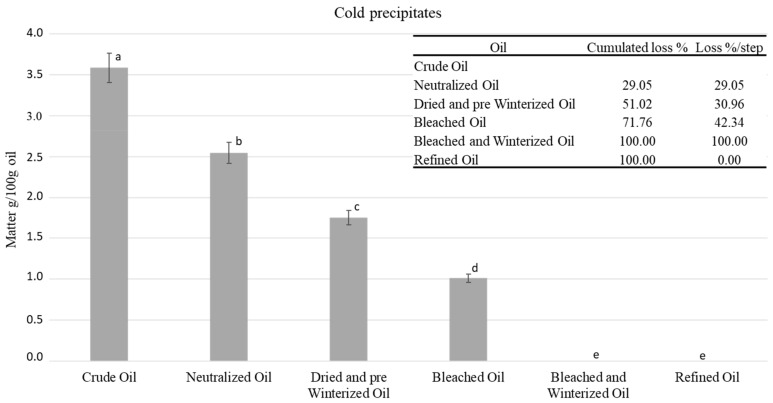
Evolution of the content of cold precipitates during the refining process, ^a–e^ different letters mean significant differences between steps *p* < 0.05 (Tukey’s test).

At first, we were interested in the physical aspects. We studied the loss in cold crystallizable matter. Figure 1 shows the results obtained from the physical analysis of the sunflower oils at different refining stages. The chemical-refining process had a significant effect and allowed us to completely eliminate the precipitates generated by cold temperatures (Figure 1). The content of cold precipitates decreased significantly from 3.6% to 0.0% in the crude and refined oil, respectively. At each refining stage, the loss percentage exceeded 29%. In terms of relative percentage, the stage of bleaching and winterizing permitted the elimination of the maximum of such precipitates due to its treatment temperatures being lower compared to those of the pre-winterization, allowing the complete precipitation of waxes, which are the main component of the cold precipitates. However, the quantities eliminated (1 g/100 g) were similar between the first stage of neutralization and the fourth stage of bleaching and winterizing. These precipitates which form crystal bodies in vegetable oil are mainly composed of waxes, which affected the weight of the oil after refining [29]. They decrease the transparency and cause a certain change in oil colors (see Section 3.3). At the same time, wax molecules are eliminated because they degrade the organoleptic qualities such as the smell, flavor and palatability of the oil. However, this does not mean that this lipidic fraction is lost; it has uses in other industrial fields [30]. Wax material is employed for making lubricants [31] and as impregnating agents of skin, leather, wood, and paper. It can be used in cosmetics [32] and in skin- and hair-care products [33,34]. It is mostly used as a structuring/thickening agent [35]. In agri-food processing, it can also be used as a fruit preservative [36] and for some food-specific applications [37]. Finally, biobased waxes may serve to replace petroleum-based products in many applications. 

### 2.2. Fatty Acids

The analysis of fatty-acid composition during the phases of the refining process show that there was no significant evolution of the composition during the several refining steps (Table 1). In terms of fatty-acid composition, nutritional quality was preserved. The same results were obtained for high-oleic sunflower oil (data not shown). Several works showed no significant differences in fatty-acid composition due to the refining conditions [38,39]. Such differences would be due to the absence of undesirable polymerization of lipidic molecules during the refining process.

### 2.3. Colorimetry

Carotenoids were present in the crude oil at 19.7 mg/kg oil (Table 2), which are lower quantities than the ones found in the literature [37]. Then the content is significantly and drastically reduced with a loss of 63.8% during the first step of the refining process, and this loss is continued until almost no carotenoids remain in the refined oil with a global loss of 98.6%; the final content of 0.5 µg/g of oil that was determined is close to the values from the literature [40,41]. Carotenoids present significant health benefits and important techno-functional properties for applications in the agri-food industries. Thanks to their provitamin-A activity and antioxidant properties, carotenoids have received great attention [42]. Therefore, industrial refining processes should preserve as much of these molecules as possible for their antioxidant capacity and their role in the protection of unsaturated fatty acids. According to the literature, the antioxidant activity depends on the concentration of carotenoids in edible oils. When low concentrations of beta-carotene (<30 ppm) were added to sunflower oil no effect was found [43], while at concentrations between 50 and 300 ppm carotenoids were pro-oxidant, and quantities above 300 ppm had an antioxidant action [44]. Therefore, high concentration of carotenoids should be maintained in edible oil in order to retain their protective and antioxidative role. The refined oil in our study exhibited a concentration of 500 ppm, which could be sufficient for fatty-acid protection if other antioxidant molecules are also present in the oil [43].

Chlorophylls present in the crude oil at a concentration 0.31 mg/100 g of oil were only partially eliminated up to 70% in the successive stages, reaching in the final step a concentration of 0.09 mg/100 g of oil.

The chlorophyll and carotenoid pigments present in crude vegetable oils are responsible for the greenish color. The oil color is considered as one of the most significant sensory properties evaluated by consumers, as well as a crucial quality index. Chlorophyll is thermally decomposed into another pigment pheophytin, which leads to a dull, dark-colored oil. Direct oxidation of these pigments and or interactions of oxidized triglycerides with carotenoid compounds may lead to oil darkening [45]. Pigments contribute to an off-flavor and may also act as photosensitizers and catalyze the oxidation of the vegetable oil in the presence of light, thus diminishing its storage stability [46]. Therefore, one of the major industrial concerns in the refining process is to diminish pigment molecules in crude oils. 

Reductions in carotenoids, which comprise carotenes, chlorophyll and waxes, during the refining stages impact oil color. The color observed in the samples before refining showed a higher lightness (L) with a more green and yellow profile. After the degumming, the color was paler and more balanced (close to zero values). The color intensity was also decreased. The values obtained were not as high as those found in the literature [47].

As shown in Table 2, the lightness increased significantly from 86.6 in crude oil to 98.5 in refined oil, recording a cumulated increase of 12%. The neutralization step exhibited the greatest increase percentage of about 5%. Correlation studies showed that the lightness is significantly and highly negatively correlated with the content of cold precipitates (r^2^ = 0.90). This means that molecules with a low cold solubility (waxes and others) would be responsible for the turbidity of the vegetable oils.

The redness declined gradually as a function of the refining stages. It reduced from −1.34 in crude oil to −1.2 in refined oil. The yellowness decreased from 39.0 to 25.6 over the entire refining process. The neutralization step significantly reduced the values of a and b. It exhibited the highest reduction followed by the second step, drying and pre-winterizing. Bleaching is recognized to be the most important stage for eliminating color (due to activated bleaching earths). In other studies on vegetable oils, the color was found to be completely removed after the deodorization step, most probably due to the degradation of color compounds and highly unsaturated molecules under the effect of high temperature [48,49,50]. Besides, changes in color were correlated with the reduction in cold precipitates containing undesirable compounds such as FFA, phospholipids, and waxes (Figure 1). Therefore, a large fraction of pigments was removed with gum matter. 

### 2.4. Tocopherol Analysis

The tocopherol content in the crude samples varied from 730 to 775 mg/kg of oil, which were equivalent to values found in the literature [51,52] (Table 3). In refined oil the total quantity of tocopherols varied from 680 to 720 µg/g of oil, which were higher than those in the literature [48,52,53]. The differences could be explained by the variety used and the environmental conditions during sunflower growth [54]. Differences in refining conditions could also explain the final amount of tocopherols in the refined oil. The main loss was encountered during the neutralization step where there was a mean significant loss of 7.2% of the total content, whereas in the literature the main loss was found in the deodorization step and reached cumulated losses of 24% [52,53]. The bleaching step also highly reduced the tocopherol content by 7.1%, whereas during the others stages the reduction was not significant (Table 3). In the residual bleaching earths, tocopherols were analyzed (data not shown), and they were present (but were not quantified). The US Institute of Medicine recommends a dietary daily intake of vitamin E between 12 and 15 mg/day for adults [55]. α-tocopherol is the isomer with the highest vitaminic activity and sunflower oil is the oil with the highest proportion on this isomer [56], which was also the case in the oils analyzed here, representing 99% of the total content with no evolution of that percentage during the refining process (data not shown). The tocopherols lost in the process could be recovered and easily purified to be used in sunscreens and cosmetics [57] due to the benefits of their antioxidant properties [58]. Future goals would be to increase the initial content of tocopherols in the sunflower oil by the selection of varieties with high tocopherol content [59] and/or by reducing the global losses to less than 10% during the industrial process.

### 2.5. Phytosterol Analysis

Phytosterol content was decreased from 273 mg/100 g of oil in crude oil to a mean of 221 mg/100 g in refined oil. These values are in accordance with those in the literature [51,60] (Table 3). Significant losses were encountered during neutralization (8.8% of the total) probably linked to the association of sterols to soaps [61]. Similar to what was observed with the tocopherols, phytosterols were also present in the bleaching earths (data not shown). This was followed by significant losses during the drying and winterization steps; the total losses were lower than those previously found in the literature, which reached up to 50% [62], but the refining process studied by the authors, in our point of view, is obsolete, and/or their process conditions were not optimized (temperature, pression, time, etc.). Over recent decades, there have been improvements made in industrial oil refining in order to preserve the oil quality and as a result, losses have been minimized. Phytosterols have already proven their efficacity in reducing LDL-cholesterol in blood [63] and reducing the risk of cancer [64]. They are important for health and the European Food Safety Authority has recently extended the use of phytosterol as a novel and safe food additive [65]. For these reasons, it is necessary that the refining process retains the maximum quantities of phytosterols in the oil. Besides, there could be an economical interest to extracting these sterols from waste products in order to use them in supplemented foods [66]. Already-developed cultivars with a high phytosterol content [67] should be largely used and improved in order to increase the content of those molecules in refined commercial oils.

### 2.6. Squalene Analysis

Squalene content in crude oil reached 17.68 mg/100 g of oil, values which are comparable to those found in the literature [68]. Almost half (45%) was eliminated in the sequence of stages during the refining, neutralization and winterization steps, which had the most effect on the reduction in the total content of squalene (Table 3). Squalene from vegetal origin has started to be of interest [69] as a dietary supplement and nutraceutical. Evaluating the capacity to recover the eliminated squalene in the neutralization and bleaching residues of sunflower-oil refinement will allow the obtention of an extract of high purity that could be of potential economic interest for industries as a high-value by-product. Another option could be to increase the content of squalene in sunflower oil by breeding techniques, but to our knowledge there has been no research on this topic yet.

### 2.7. Antioxidant Activity

#### 2.7.1. Reducing-Capacity Analysis

**Figure 2:**
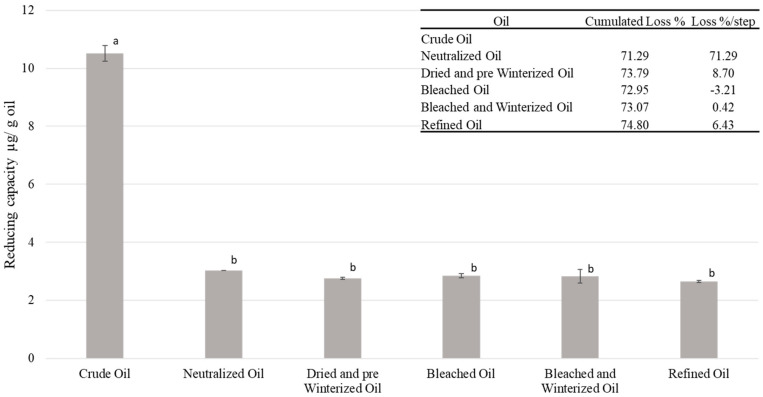
Evolution of the reducing capacity while refining the oil, ^a,b^ different letters mean significant differences between steps *p* < 0.05 (Tukey’s test).

Reducing capacity during the refining phases was mainly eliminated in the first step of the refining process with a reduction of up to 66%, whereas the content remained stable in the following stages (Figure 2). The oxidation tendency of reducing molecules under alkaline conditions could explain their loss during the neutralization treatment [70]. The industrial process is meant to reduce these molecules, mainly polyphenols, as they can generate unfavorable effects during the subsequent steps such as saturation of the bleaching earths [71] or induction of oil browning during deodorization, thereby altering the oil flavor and the appearance [72]. The measured concentrations agree with those found in the literature [73]. The level of reduction is equivalent to the adsorption level encountered in several clays that could be used during the bleaching step [24]. Unfortunately, this loss partially reduces the health interest of this oil. Indeed, a diet rich in polyphenols may reduce the risk of several chronic diseases [74]. That said, the initial reducing-molecule (polyphenols) content in oils is significantly low compared to other sources of polyphenols such as coffee or tea, and oils could only play a minimal role in this health aspect.

#### 2.7.2. DPPH

**Figure 3:**
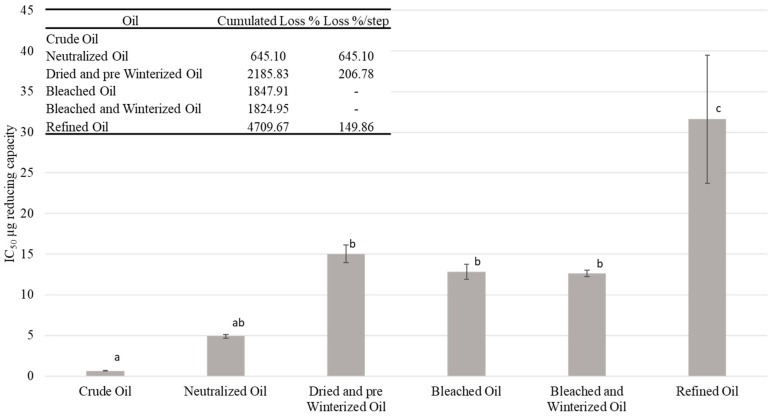
Evolution of the stability of the oil over the main steps of the refining process measured by the DPPH IC_50_, ^a–c^ different letters mean significant differences between steps *p* < 0.05 (Tukey’s test).

Figure 3 shows the evolution of the IC_50_ as oil refinement progressed. This index reflects the quantity of the product necessary to reduce by 50% the activity of DPPH; the higher the activity of antioxidants, the lower the value of the index [75]. The oil after the refining stage seemed less stable compared to the crude oil in terms of oxidation. The value of IC_50_ evolved from nearly zero to more than 30, with the largest increases during the drying and pre-winterizing step and the final stage. Antioxidant activity decreased when the IC_50_ increased. The cumulated losses were about 4.709% in the refined oil. These losses in antioxidant activity were directly correlated to the losses in reducing capacity (including polyphenols).

### 2.8. Oil Stability

**Figure 4:**
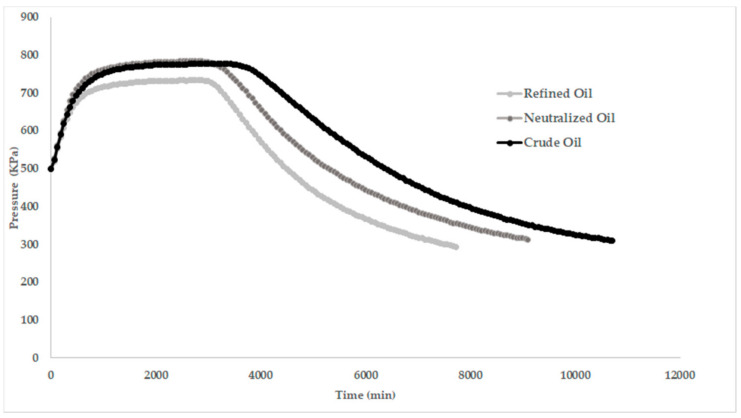
Oxidation stability of oil over time at different steps of the refining process.

Figure 4 shows the oxidation curves obtained by the RapidOxy^®^ method, wherein it is generally recognized that at the accelerated stage, the oxidation rate mainly depends on oxygen availability and temperature [76]. The results show that oxidative stability decreased as a function of the refining stages. The crude oil was the most stable while the refined oil showed the lowest oxidative stability. This demonstrates that oil composition was changed as a function of the refining steps. Otherwise, antioxidant molecules are lost during refinement along with undesirable molecules, as confirmed by several authors in different vegetable oils [49,50,77,78]. 

## 3. Materials and Methods

### 3.1. Samples

LSO samples were collected at 6 different stages of an industrial refining process. The refining process is a combination of physical (bleaching, winterization and deodorization) and chemical methods. The neutralization step was performed with an alkaline solution at 85 °C. Then it was washed with clear water at 95 °C and eliminated by centrifugation. The oil was then dried at 100 °C to completely eliminate the water, after which the oil was cooled to 15 °C. The bleaching was performed at 90 °C. The winterization was performed at 5–8 °C and the filtering was performed at 15–18 °C. The deodorization process was carried out under vacuum at 180 °C. Samples were collected after each step: 1—crude oil; 2—neutralized oil; 3—dried and pre-winterized oil; 4—bleached oil; 5—bleached and winterized oil; 6—refined oil. The samples were kept away from the light, high temperature and oxygen to avoid auto-oxidation.

### 3.2. Reagents

All reagents were of analytical grade. Reagents and standards were purchased from Sigma-Aldrich (St. Quentin Fallavier, France). Redistilled water was used for preparation of solutions. 

### 3.3. Physical Analyses

#### 3.3.1. Cold Precipitates

Cold precipitates were analyzed in order to evaluate the visual aspect of the oil at each step of the refining process. Oil samples were weighed into tubes and kept for 24 h at 4 °C. Then, tubes were centrifuged at 12,500× *g* for 15 min at 4 °C. Supernatants were discarded and the pellets, mainly constituted of waxes, were kept and weighed. Results were expressed in terms of g of pellet per 100 g of oil.

#### 3.3.2. Color Analysis

Oil-sample color was determined using a Bench-top Colorimeter CR-5 (Konica Minolta, Roissy en France, France). An automatic white calibration (reflectance)/100% calibration (transmittance) using an internal white-calibration plate was performed prior to the collection of experimental measurements. Color measurements were performed in triplicate according to instrument operation guidance. L*, a*, and b* parameters were recorded for each oil sample. L* indicates lightness, a* is the red/green coordinate, and b* is the yellow/blue coordinate.

### 3.4. Chemical Analysis

#### 3.4.1. Fatty Acids Composition

Fatty-acid profile was established by transmethylation of triglycerides and further analysis by gas chromatography [79]. Forty (40) mg of oil were transmethylated in 900 µL of diethyl ether with 100 μL of tetramethyl ammonium hydroxide (in 25% methanol). The mix was incubated at room temperature for 10 min. Focus gas chromatography (ThermoFisher Scientific, Courtaboeuf, France) with a flame ionization detector (FID) was used for fatty-acid quantitation. Fatty-acid separation was performed using a capillary BPX-90 column (30 m × 0.25 mm × 0.25 μm; Trajan Scientific Europe Ltd., Milton Keynes, UK). One (1) μL of sample was injected at 270 °C with a split ratio of 1/14. Oven temperature was set at 170 °C for 5 min and was increased to 230 °C with a 45 °C /min slope. The temperature was maintained for 2 min. Hydrogen was used as an eluent at a flow rate of 0.7 mL/min. Detection was performed at 280 °C.

#### 3.4.2. Tocopherols Analysis

Forty (40) mg of each oil sample were dissolved into 1 mL of iso-octane. Analyses were performed using a surveyor system coupled to fluorometer detector (Thermo Fisher Scientific Corporation, Courtaboeuf, France). The excitation wavelength was set at 298 nm while signal was recorded at the emission wavelength of 344 nm. Tocopherols were separated in an Acclaim™ C30 analytical column, 150 × 4.6 mm, particle size 5 µm (Thermo Fisher Scientific Corporation, Courtaboeuf, France) connected in series with a pre-column with the same characteristics. The column was maintained at 30 °C during the run. Solvent A was methanol, solvent B acetonitrile and solvent C tert-butyl methyl ether. The flow rate was 1 mL/min and the injection volume was set at 3 µL. The gradient profile was as described in Table 4. The identification was based on retention time, and the quantification of tocopherols was carried out using external standards of the individual tocopherols. Results were expressed as mg of tocopherols per kg of oil.

#### 3.4.3. Phytosterols and Squalene Analysis

Sterol extraction and GC analysis were conducted as previously described [51]. Determination of sterol content followed the normalized procedure [80]. About 250 mg of oil and 200 µL of a freshly prepared solution of betulin (1 mg/mL acetone), as an internal standard, were subjected to saponification by adding 1 mL of ethanolic KOH (0.5 M) followed by incubation at 100 °C for 15 min. Saponification was stopped by adding 1 mL of ethanol. The solution was transferred to a glass column containing 2 g of aluminum oxide (Sigma-Aldrich, St. Quentin Fallavier, France) soaked with ethanol. In order to recover the unsaponifiable matter, the column was washed with 5 mL of ethanol and 30 mL of diethyl ether into a new balloon. Solutions were concentrated using a rotavapor system and recovered with 2 mL of pyridine. Unsaponifiable matter (900 µL) was derivatized using 100 μL N-methyl-N (trimethylsilyl)-heptafluorobutyramide (5:95 *v*/*v*) and heated for 15 min at 105 °C in an oil bath. Analyses were performed using Trace 1310 GC coupled with mass spectrometer (MS) detector ISQ 7000 (Thermo Fisher Scientific Corporation, Courtaboeuf, France). Splitless injections were performed with 1 μL sample volume. Three minutes after injection, the split valve was opened. Separations were achieved with a Phenomenex fused silica capillary ZB-5 inferno (30 m × 0.25 mm × 0.25 μm, Paris, France). Helium carrier-gas flow rate was set at 0.7 mL/min. The injector was set at 320 °C and the transfer tube and source were set at 250 °C and 200 °C, respectively. The initial temperature of the GC oven was 240 °C and increased to 320 °C at 4 °C/min, then was held for 10 min until it decreased to initial conditions. The identification of molecules was based on their MS specter and retention time of standards and with the relative times to betulin given in the norm [80]. The calibration results were based on the integrated areas. Analyses were performed in triplicate.

#### 3.4.4. Reducing Capacity

Reducing-capacity molecules, mainly phenolic compounds, were extracted from sunflower oil (20 g) using 20 mL of a methanolic solution (80%, *v*/*v*). The reducing capacity in methanolic extracts was determined spectrophotometrically using the Folin–Ciocalteu protocol. Briefly, 0.1 mL of each sunflower-oil reducing extract was taken in a centrifuge tube. Folin–Ciocalteu’s reagent (0.1 mL) was added and the mixture was thoroughly vortexed. After 10 min, 0.7 mL of sodium carbonate (7.5%) was added to each sample. Then, tubes were vortexed thoroughly after adding distilled water (8 mL). Tubes were kept for 1 h at room temperature in the dark and centrifuged for 2 min at 8000× *g*. The absorbance was read at 725 nm (Evolution™ 201 spectrophotometer, ThermoFisher Scientific, Courtaboeuf, France) using methanol 80% as a blank for background subtraction. Calibration curves were obtained using different concentrations of gallic-acid solutions. Three calibration curves were plotted using the least-squares method resulting in R^2^ = 0.999, RSD% ≤ 5%. Reducing capacity was calculated and expressed as milligrams of gallic-acid equivalents (mg GAE) per 100 g of oil.

#### 3.4.5. Pigments Analysis

The total carotenoid content was determined according to the PORIM-test method [81] and the procedure previously described by Rodriguez-Amaya et al. [82]. About 1.5 g of sunflower-oil samples were dissolved in 10 mL of n-hexane. Then, the absorbance at 450 nm was measured against n-hexane using an Evolution™ 201 spectrophotometer (ThermoFisher Scientific, Courtaboeuf, France) in a 1 cm quartz cell. Quantitative calibration curves were established using solutions of β-carotene at concentrations ranging from 0.10 to 4 µg/mL. The total carotenoid content was expressed as mg of β-carotene equivalents per kg of oil.

The content of chlorophylls was estimated according to the AOCS method [83]. The absorbance of sunflower-oil samples was measured at 630, 670, and 710 nm using an Evolution™ 201 spectrophotometer (ThermoFisher Scientific, Courtaboeuf, France) and expressed as mg of pheophytin per kg of oil.

#### 3.4.6. Antioxidant Properties

##### DPPH Analysis

The antioxidant capacity of sunflower oils from various stages of the refining process was determined according to the DPPH (2,2-diphenyl-1-picrylhydrazyl) method as previously described [84]. Each methanolic extract (1 mL) of sunflower oil (or BHT standard solutions at different concentrations) was mixed with 1 mL of methanol 80% (*v*/*v*) and 0.5 mL of DPPH methanolic solution (35.5 mg/L). The mixture was vigorously vortexed and left in darkness for 1 h at room temperature. The absorbance was measured at 532 nm against methanol 80% as a blank solution using an Evolution™ 201 spectrophotometer (ThermoFisher Scientific, Courtaboeuf, France). The scavenging of DPPH (%) was determined as follows:

Percentage inhibition (%) = [(A0 − A1)/A0)] × 100

where A0 is the absorbance of the control and A1 the absorbance of the extract. The results are expressed in terms of the IC_50_ value, which is the effective concentration at which the antioxidant activity is 50%. All experiments were performed in triplicate.

##### Accelerated Oxidative Stability

Several accelerated oxidation tests (Rancimat test and Shaal test) were already used for evaluation of the oxidative stability [85]. Thanks to its important advantages such as time savings and a small sample volume, the oxidative stability of the oil over the course of the refining process was studied using RapidOxy^®^ device (Anton Paar, Les Ulis, France). It is based on the increase in oxygen pressure and temperature inside a sealed chamber, allowing determination of the oxidative stability of the oils. In recent years, the RapidOxy^®^ test has become more relevant. 3 mL of the sunflower-oil sample were analyzed according to the manufacturer’s protocol. Temperature and pressure were set to 120 °C and 750 kPa, respectively, and the desired conditions were adapted from [86]. Then, the tank was sealed and the oxygen pressure was recorded as a function of time. The test was stopped after 60% of the oxygen had pressure dropped. 

### 3.5. Statistical Analysis

All experiments were performed in triplicate and the experimental data were reported as means ± standard deviation. Each determination was performed at the same time for the sunflower oil samples. The normality of the results was tested, then an analysis of variance (ANOVA) and Tukey’s post-hoc multiple-range test (α = 0.05) were performed on the experimental data using XLSTAT software (Addinsoft SARL, New York, NY, USA).

## 4. Conclusions

In this study, LSO samples were collected at six different stages of the industrial refining process and analyzed. The results showed a significant effect of the refining steps on the molecules of interest (i.e., tocopherols, phytosterols, squalene, reducing capacity, carotenoids). The neutralization step was the most influential in reducing the content of all the studied parameters. Consequently, oxidant stability was impacted by the reduction in molecules with antioxidant activity. The losses in the antioxidant activity were the most important.

It is undeniable that the industrial oil-refining process is performed to maintain the quality of the oil suitable for human consumption for as long as possible. This involves making the oil tasteless, odorless, changing its color and modifying its crystalline appearance. Preserving the health benefits of consuming oil should also guarantee its nutritional quality. The oil analyzed in this work maintains a high level of nutrients that are of interest to health benefits. Further optimizations of the refining process and breeding strategies to highly increase the starting concentrations of these antioxidant molecules should be encouraged in order to provide consumers with significant amounts of antioxidants, which may help to reduce some population health issues.

## Figures and Tables

**Table 1:** Evolution of the content of the fatty acids during the oil-refining process. Means ± standard deviations followed by different superscript letters within the same column are significantly different according to Tukey’s test. All experiments were performed in triplicate. CV: Coefficient of variation.

	Fatty Acids			
	C16: 0	C18: 0	C18: 1	C18: 2
Crude	6.74 ± 0.21 ^a^	3.80 ± 0.33 ^a^	26.34 ± 0.22 ^a^	61.58 ± 0.19 ^a^
Neutralized	6.64 ± 0.22 ^a^	3.71 ± 0.15 ^a^	26.96 ± 0.45 ^a^	61.87 ± 0.39 ^a^
Dried & pre-Winterized	6.73 ± 0.30 ^a^	3.72 ± 0.19 ^a^	27.25 ± 0.51 ^a^	61.44 ± 0.56 ^a^
Bleached	6.77 ± 0.11 ^a^	3.72 ± 0.25 ^a^	26.75 ± 0.26 ^a^	61.96 ± 1.23 ^a^
Bleached & Winterized	6.83 ± 0.12 ^a^	3.73 ± 0.17 ^a^	26.51 ± 0.52 ^a^	62.07 ± 0.31 ^a^
Refined	6.85 ± 0.08 ^a^	3.73 ± 0.10 ^a^	28.06 ± 1.35 ^a^	60.45 ± 1.08 ^a^
CV	1.12	0.87	2.29	0.96

^a^ Means ± standard deviations followed by different superscript letters within the same column are significantly different according to Tukey’s test (*p* < 0.05). All experiments were performed in triplicate. CV: Coefficient of variation.

**Table 2:** Evolution of the color of the oil during the refining process measured in terms of total carotenoid content, chlorophylls and the CIE L*a*b* color space.

Refining Oil Steps	Total Carotenoids (mg/kg Oil)			Chlorophylls (mg/100 g Oil)			Colorimetry		
	Content	Cumulated Loss (%)	Loss (%)/ Step	Content	Cumulated Loss (%)	Loss (%)/ Step	L*	a*	b*
Crude Oil	19.70 ± 0.31 ^a^		-	0.31± 0.03 ^a^		-	86.58 ± 0.17 ^a^	−1.34 ± 0.15 ^ac^	39.04 ± 0.52 ^a^
Neutralized Oil	7.11 ± 0.79 ^b^	63.80	63.80	0.24 ± 0.10 ^b^	22.58	22.58	91.54 ± 0.2 ^b^	−2.81 ± 0.22 ^a^	33.76 ± 0.91 ^b^
Dried and pre-Winterized Oil	2.46 ± 0.02 ^c^	87.50	65.40	0.20 ± 0.02 ^bc^	22.58	=	95.41 ± 0.70 ^c^	−4.76 ± 0.17 ^b^	34.69 ± 0.35 ^b^
Bleached Oil	1.51 ± 0.10 ^cd^	92.30	38.60	0.17 ± 0.02 ^cd^	45.16	29.16	96.51 ± 0.3 ^c^	−4.43 ± 0.09 ^b^	25.40 ± 0.99 ^c^
Bleached and Winterized Oil	1.37 ± 0.00 ^d^	93.04	9.10	0.12 ± 0.02 ^de^	61.29	29.41	96.88 ± 0.15 ^c^	−4.65 ± 0.10 ^b^	27.68 ± 1.29 ^c^
Refined Oil	0.28 ± 0.05 ^e^	98.57	79.30	0.09 ± 0.02 ^e^	70.96	25.00	98.46 ± 0.11 ^d^	−1.20 ± 0.06 ^c^	25.60 ± 0.25 ^c^

^a–e^ Means ± standard deviations followed by different superscript letters within the same column are significantly different according to Tukey’s test (*p* < 0.05).

**Table 3:** Content of tocopherol, phytosterol and squalene (Mean ± Standard deviation) and their losses found at each step of the refining process for the two types of samples, solvent-extracted oil and cold-pressed oil.

Refining Oil Steps	Total Tocopherol (mg/kg Oil)			Total Phytosterol (mg/100 g Oil)			Squalene (mg/100 g Oil)		
	Content	Cumulated Loss (%)	Loss (%)	Content	Cumulated Loss (%)	Loss (%)	Content	Cumulated Loss (%)	Loss (%)
Crude	764.82 ± 23.08 ^a^		-	274.78 ± 4.05 ^a^		-	17.68 ± 0.08 ^a^		-
Neutralized	704.80 ± 15.21 ^b^	7.85	7.86	250.57 ± 9.75 ^b^	8.81	8.81	13.68 ± 0.68 ^b^	22.60	22.60
Dried and pre-Winterized	703.78 ± 8.14 ^b^	8.00	=	233.36 ± 1.01 ^cd^	15.08	6.87	13.08 ± 0.41 ^b^	26.47	=
Bleached	674.16 ± 15.16 ^b^	11.85	4.21	237.24 ± 2.73 ^c^	13.66	=	10.25 ± 1.82 ^cd^	42.02	21.60
Bleached and Winterized	693.07 ± 22.84 ^b^	9.38	=	235.70 ± 2.72 ^c^	14.22	=	11.83 ± 0.24 ^bc^	33.10	=
Refined	699.67 ± 20.56 ^b^	8.51	=	221.12 ± 2.37 ^cd^	19.53	6.20	9.72 ± 0.12 ^d^	45.02	17.80

^a–d^ Means ± standard deviations followed by different superscript letters within the same column are significantly different according to Tukey’s test (*p* < 0.05).

**Table 4:** Gradient solvents for tocopherol chromatography separation.

Time	Flow mL/min	% Methanol	% Acetonitrile	% Tert-Butyl Methyl Ether
0	1	75	25	0
10	1	70	25	5
20	1	55	25	20
35	1	35	15	50
38	1	35	15	50
39	1	75	25	0
40	1	75	25	0

## Data Availability

Not applicable.

## References

[1] FAOSTAT. http://www.fao.org/faostat/en/#home.

[2] WHO/FAO (1994). Fats and Oils in Human Nutrition. Report of a Joint FAO/WHO Expert Consultation.

[3] FAO (1999). SECTION 2. Codex Standards for Fats and Oils from Vegetable Sources.

[4] Neto A.R., Rauen A., Mourad A., Henriques E., Alves R.M.V. (2016). Environmental Effect on Sunflower Oil Quality. Crop Breed. Appl. Biotechnol..

[5] Ayerdi Gotor A., Berger M., Labalette F., Centis S., Daydé J., Calmon A. (2015). Comparative Analysis of Fatty Acids, Tocopherols and Phytosterols Content in Sunflower Cultivars (*Helianthus annuus*) from a Three-Year Multi-Local Study. Phyton Int. J. Exp. Bot..

[6] Schulte L.R., Ballard T., Samarakoon T., Yao L., Vadlani P., Staggenborg S., Rezac M. (2013). Increased Growing Temperature Reduces Content of Polyunsaturated Fatty Acids in Four Oilseed Crops. Ind. Crops Prod..

[7] Matthäus B. (2007). Use of Palm Oil for Frying in Comparison with Other High-Stability Oils. Eur. J. Lipid Sci. Technol..

[8] Labalette F., Jouffret P., Merrien A. Oleic Sunflower Production: Current Situation and Trends for the Future. Proceedings of the XVIIIth International Congress.

[9] Gondé P., Morin O. (2012). Optimization of oil for industrial frying process: Mc Cain’s testimony. OCL—Oilseeds Fats.

[10] Leao J.D., Bouillon V., Muntada L., Johnson C., Wilson P., Vergnes O., Dano C., Igartua A., Mendoza G. (2016). New Formulations of Sunflower Based Bio-Lubricants with High Oleic Acid Content—VOSOLUB Project. OCL.

[11] Ramos M.J., Fernández C.M., Casas A., Rodríguez L., Pérez Á. (2009). Influence of Fatty Acid Composition of Raw Materials on Biodiesel Properties. Bioresour. Technol..

[12] Cicerale S., Liem D.G., Keast R. (2016). Consumer Perception, Attitudes, Liking and Preferences for Olive Oil. Products from Olive Tree.

[13] Packer L., Landvik S. (1989). Vitamin E: Introduction to Biochemistry and Health Benefits. Ann. N. Y. Acad. Sci..

[14] Ogbe R.J., Ochalefu D.O., Mafulul S.G., Olaniru O.B. (2015). A Review of Dietary Phytosterols: Their Occurences, Metabolism and Health Benefits. Asian J. Plant Sci. Res..

[15] Kamal-Eldin A. (2006). Effect of Fatty Acids and Tocopherols on the Oxidative Stability of Vegetable Oils. Eur. J. Lipid Sci. Technol..

[16] Pehlivan E., Arslan G., Gode F., Altun T., Musa Özcan M. (2008). Determination of Some Inorganic Metals in Edible Vegetable Oils by Inductively Coupled Plasma Atomic Emission Spectroscopy (ICP-AES). Grasas Aceites.

[17] Hamilton R.J., Kalu C., Prisk E., Padley F.B., Pierce H. (1997). Chemistry of Free Radicals in Lipids. Antioxid. Food.

[18] Rodrigues M.S., Dos Passos R.M., de A Pontes P.V., Ferreira M.C., Meirelles A.J.A., Stevens C.V., Maximo G.J., Sampaio K.A. (2021). Enzymatic Degumming of Rice Bran Oil Using Different Commercial Phospholipases and Their Cocktails. Life.

[19] Abedi E., Amiri M.J., Sahari M.A. (2020). Kinetic, Isotherm and Thermodynamic Investigations on Adsorption of Trace Elements and Pigments from Soybean Oil Using High Voltage Electric Field-Assisted Bleaching: A Comparative Study. Process. Biochem..

[20] Maszewska M., Florowska A., Dłużewska E., Wroniak M., Marciniak-Lukasiak K., Żbikowska A. (2018). Oxidative Stability of Selected Edible Oils. Molecules.

[21] Baştürk A., Boran G., Javidipour I. (2017). Effects of Ascorbyl Palmitate and Metal Ions on Oxidation of Sunflower Oil under Accelerated Oxidation Conditions. J. Anim. Plant Sci..

[22] Kalua C.M., Allen M.S., Bedgood D.R., Bishop A.G., Prenzler P.D., Robards K. (2007). Olive Oil Volatile Compounds, Flavour Development and Quality: A Critical Review. Food Chem..

[23] Baümler E.R., Carrín M.E., Carelli A.A. (2016). Extraction of Sunflower Oil Using Ethanol as Solvent. J. Food Eng..

[24] El-Hamidi M., Zaher F. (2016). Comparison Between Some Common Clays as Adsorbents of Carotenoids, Chlorophyll and Phenolic Compounds from Vegetable Oils. Am. J. Food Technol..

[25] Brevedan M.I.V., Carelli A.A., Crapiste G.H. (2000). Changes in Composition and Quality of Sunflower Oils during Extraction and Degumming. Grasas Aceites.

[26] Jung M.Y., Yoon S.H., Min D.B. (1989). Effects of Processing Steps on the Contents of Minor Compounds and Oxidation of Soybean Oil. J. Am. Oil Chem. Soc..

[27] Ayerdi Gotor A., Rhazi L. (2016). Effects of Refining Process on Sunflower Oil Minor Components: A Review. OCL.

[28] Zhang Q., Saleh A.S.M., Chen J., Shen Q. (2012). Chemical Alterations Taken Place during Deep-Fat Frying Based on Certain Reaction Products: A Review. Chem. Phys. Lipids.

[29] Aluyor E., Ozigagu C., Oboh I. (2009). Chromatographic Analysis of Vegetable Oils: A Review. Sci. Res. Essay.

[30] Fei T., Wang T. (2017). A Review of Recent Development of Sustainable Waxes Derived from Vegetable Oils. Innov. Food Sci. Foodomics Technol..

[31] Shalini T., Martin A. (2020). Identification, Isolation, and Heterologous Expression of Sunflower Wax Synthase for the Synthesis of Tailored Wax Esters. J. Food Biochem..

[32] Chalapud M.C., Baümler E.R., Carelli A.A. (2017). Characterization of Waxes and Residual Oil Recovered from Sunflower Oil Winterization Waste. Eur. J. Lipid Sci. Technol..

[33] Maru A., Pattamatta U., Patravale V. (2009). Sunflower Wax as a New Natural Cosmetic Raw Material: Purification and Application in Lipsticks. Res. J. Pharm. Dos. Forms Techonol..

[34] Maru A., Lahoti S. (2018). Formulation and Evaluation of Moisturizing Cream Containing Sunglower Wax. Int. J. Pharm. Pharm. Sci..

[35] Winkler-Moser J.K., Anderson J., Byars J.A., Singh M., Hwang H.-S. (2019). Evaluation of Beeswax, Candelilla Wax, Rice Bran Wax, and Sunflower Wax as Alternative Stabilizers for Peanut Butter. J. Am. Oil Chem. Soc..

[36] Soomro K.R., Sherazi T.H., Shaikh S.A. (2013). Effects of Sunflower Wax Coating on Physicochemical Changes of *Mangifera indica* L. in Storage Life. Pak. J. Anal. Environ. Chem..

[37] Demirci M., Pehlivanoğlu H. Potential Food Applications of Sunflower Wax. Proceedings of the International Sunflower Conference.

[38] Mohdaly A., Seliem K., EL-Hassan A., Mahmoud A. (2017). Effect of Refining Process on the Quality Characteristics of Soybean and Cotton Seed Oils. Int. J. Curr. Microbiol. Appl. Sci..

[39] Pal U.S., Patra R.K., Sahoo N.R., Bakhara C.K., Panda M.K. (2015). Effect of Refining on Quality and Composition of Sunflower Oil. J. Food Sci. Technol..

[40] Buhalova D., Nikolova K., Antova G., Milkova-Tomova I., Aladjadjiyan A., Aleksieva Y., Petkova Z. (2014). Comparative Characteristics of Sunflower Oil with Supplement of Traditional Bulgarian Herbs. Bulg. Chem. Commun..

[41] Vrbikova L., Schmidt S., Kreps F., Tmáková L., Certik M., Sekretár S. (2014). Degradation of Selected Nutrients in Sunflower Oils during Long-Term Storage. Czech J. Food Sci..

[42] Aguilar-Espinosa M., Alcalde M., Alonso G., Álvarez R., Angaman D., Ahrazem O., Avalos J., Bagur M., Benitez A., Berman J., Meléndez-Martínez A.J., Gómez L.G., Alonso B.O., Pérez-Gálvez A., Hornero-Méndez D. (2017). Carotenoides En Agroalimentación y Salud.

[43] del Pilar Sánchez-Camargo A., Gutiérrez L.-F., Vargas S.M., Martinez-Correa H.A., Parada-Alfonso F., Narváez-Cuenca C.-E. (2019). Valorisation of Mango Peel: Proximate Composition, Supercritical Fluid Extraction of Carotenoids, and Application as an Antioxidant Additive for an Edible Oil. J. Supercrit. Fluids.

[44] Zeb A. (2011). Effects of β-Carotene on the Thermal Oxidation of Fatty Acids. Afr. J. Biotechnol..

[45] Geleneksel D., Tüketilmeyen O., Sudan Ü., Ve Y., Etkisi S., Mariod A., Matthäus B., Eichner K., Hussein I. (2012). Effects of Deodorization on the Quality and Stability of Three Unconventional Sudanese Oils. GIDA Derg. J. Food.

[46] Naebi M., Torbati M., Azadmard-Damirchi S., Siabi S., Savage G.P. (2022). Changes in Physicochemical Properties of Cold Press Extracted Oil from Balangu (*Lallemantia peltata*) Seeds during Storage. J. Food Compos. Anal..

[47] Diosady L.L. (2005). Chlorophyll Removal from Edible Oils. Int. J. Appl. Sci. Eng..

[48] Suliman T.E., Meng Z., Li J.W., Jiang J., Liu Y. (2013). Optimisation of Sunflower Oil Deodorising: Balance between Oil Stability and Other Quality Attributes. Int. J. Food Sci. Technol..

[49] Chew S.-C., Tan C.-P., Long K., Nyam K.-L. (2016). Effect of Chemical Refining on the Quality of Kenaf (*Hibiscus cannabinus*) Seed Oil. Ind. Crops Prod..

[50] Wu Y., Zhou R., Wang Z., Wang B., Yang Y., Ju X., He R. (2019). The Effect of Refining Process on the Physicochemical Properties and Micronutrients of Rapeseed Oils. PLoS ONE.

[51] Ayerdi Gotor A., Farkas E., Berger M., Labalette F., Centis S., Daydé J., Calmon A. (2007). Determination of Tocopherols and Phytosterols in Sunflower Seeds by NIR Spectrometry. Eur. J. Lipid Sci. Technol..

[52] Alpaslan M., Tepe S., Simsek O. (2001). Effect of Refining Processes on the Total and Individual Tocopherol Content in Sunflower Oil. Int. J. Food Sci. Technol..

[53] Tasan M., Demirci M. (2005). Total and Individual Tocopherol Contents of Sunflower Oil at Different Steps of Refining. Eur. Food Res. Technol..

[54] Ayerdi Gotor A., Berger M., Labalette F., Centis S., Dayde J., Calmon A. (2006). Variabilité Des Teneurs et Compositions Des Composés Mineurs Dans l’huile de Tournesol Au Cours Du Développement Du Capitule. OCL.

[55] Institute of Medicine 6 (2000). Vitamin E. Dietary Reference Intakes for Vitamin C, Vitamin E, Selenium, and Carotenoids.

[56] Schwartz H., Ollilainen V., Piironen V., Lampi A.-M. (2008). Tocopherol, Tocotrienol and Plant Sterol Contents of Vegetable Oils and Industrial Fats. J. Food Compos. Anal..

[57] Alberts D.S., Goldman R., Xu M.-J., Dorr R.T., Quinn J., Welch K., Guillen-Rodriguez J., Aickin M., Peng Y.-M., Loescher L. (1996). Disposition and Metabolism of Topically Administered A-tocopherol Acetate: A Common Ingredient of Commercially Available Sunscreens and Cosmetics. Nutr. Cancer.

[58] Kusumawati I., Indrayanto G. (2013). Natural Antioxidants in Cosmetics. Studies in Natural Products Chemistry.

[59] Ayerdi Gotor A., Berger M., Labalette F., Centis S., Dayde J., Calmon A. Estimation of Breeding Potential for Tocopherols and Phytosterols in Sunflower. Proceedings of the 17th International Sunflower Conference.

[60] Yang R., Xue L., Zhang L., Wang X., Qi X., Jiang J., Yu L., Wang X., Zhang W., Zhang Q. (2019). Phytosterol Contents of Edible Oils and Their Contributions to Estimated Phytosterol Intake in the Chinese Diet. Foods.

[61] Bai G., Ma C., Chen X. (2021). Phytosterols in Edible Oil: Distribution, Analysis and Variation during Processing. Grain Oil Sci. Technol..

[62] Karaali A. (1985). The Effects of Refining on the Chemical Composition of Turkish Sunflower Seed Oil. Fette Seifen Anstrichm..

[63] Stelmach-Mardas M., Przysławski J. (2013). Clinical Aspects of Phytosterols in Human Nutrition. Forsch. Komplementarmed. 2006.

[64] Woyengo T.A., Ramprasath V.R., Jones P.J.H. (2009). Anticancer Effects of Phytosterols. Eur. J. Clin. Nutr..

[65] Turck D., Castenmiller J., De Henauw S., Hirsch-Ernst K.I., Kearney J., Maciuk A., Mangelsdorf I., McArdle H.J., Naska A., EFSA Panel on Nutrition, N.F. and F.A. (NDA) (2020). Safety of the Extension of Use of Plant Sterol Esters as a Novel Food Pursuant to Regulation (EU) 2015/2283. EFSA J..

[66] Zawistowski J., Jones P. (2015). Regulatory Aspects Related to Plant Sterol and Stanol Supplemented Foods. J. AOAC Int..

[67] García-González A., Velasco J., Velasco L., Ruiz-Méndez M.V. (2021). Attempts of Physical Refining of Sterol-Rich Sunflower Press Oil to Obtain Minimally Processed Edible Oil. Foods.

[68] Nergiz C., Çelikkale D. (2011). The Effect of Consecutive Steps of Refining on Squalene Content of Vegetable Oils. J. Food Sci. Technol..

[69] Patel A., Mu L., Shi Y., Rova U., Christakopoulos P., Matsakas L. (2020). Novel Biorefinery Approach Aimed at Vegetarians Reduces the Dependency on Marine Fish Stocks for Obtaining Squalene and Docosahexaenoic Acid. ACS Sustain. Chem. Eng..

[70] García A., Ruiz-Méndez M.V., Romero C., Brenes M. (2006). Effect of Refining on the Phenolic Composition of Crude Olive Oils. J. Am. Oil Chem. Soc..

[71] Taylor D.R. (1993). Adsorptive Bleaching. Proceedings of the World Conference on Oilseed Technology and Utilization.

[72] Zamora R., Olmo C., Navarro J.L., Hidalgo F.J. (2004). Contribution of Phospholipid Pyrrolization to the Color Reversion Produced during Deodorization of Poorly Degummed Vegetable Oils. J. Agric. Food Chem..

[73] Siger A., Nogala-Kalucka M., Lampart-Szczapa E. (2008). The Content and Antioxidant Activity of Phenolic Compounds in Cold-Pressed Plant Oils. J. Food Lipids.

[74] Williamson G. (2017). The Role of Polyphenols in Modern Nutrition. Nutr. Bull..

[75] Molyneux P. (2003). The Use of the Stable Radical Diphenylpicrylhydrazyl (DPPH) for Estimating Antioxidant Activity. Songklanakarin J. Sci. Technol. SJST.

[76] Choe E., Min D.B. (2006). Mechanisms and Factors for Edible Oil Oxidation. Compr. Rev. Food Sci. Food Saf..

[77] Gharby S., Guillaume D., Elibrahimi M., Charrouf Z. (2021). Physico-Chemical Properties and Sensory Analysis of Deodorized Argan Oil. ACS Food Sci. Technol..

[78] Gharby S., Hajib A., Ibourki M., Sakar E.H., Nounah I., Moudden H.E., Elibrahimi M., Harhar H. (2021). Induced Changes in Olive Oil Subjected to Various Chemical Refining Steps: A Comparative Study of Quality Indices, Fatty Acids, Bioactive Minor Components, and Oxidation Stability Kinetic Parameters. Chem. Data Collect..

[79] Savoire R., Lazouk M., Van-Hecke E., Roulard R., Tavernier R., Guillot X., Rhazi L., Petit E., Mesnard F., Thomasset B. (2015). Environmental and Varietal Impact on Linseed Composition and on Oil Unidirectional Expression Process. OCL.

[80] ISO12228-1 (2014). Determination of Individual and Total Sterols Contents—Gas Chromatographic Method—Part 1: Animal and Vegetable Fats and Oils.

[81] Siew W.L., Tang T.S., Tan Y.A. (1995). PORIM Test Methods.

[82] Rodriguez-Amaya D.B. (2001). International Life Sciences Institute, OMNI (Project). A Guide to Carotenoid Analysis in Foods.

[83] Ward K., Scarth R., Daun J., Thorsteinson C. (1994). A Comparison of HPLC and Spectrophotometry to Measure Chlorophyll in Canola Seed and Oil. J. Am. Oil Chem Soc..

[84] Rasolohery C.A., Ralaibia B.E., Ayerdi Gotor A., Merlier F., Benja R., Rakotovao M., Rhazi L. (2017). Chemical Characterization and Antioxidant Potential of Athroisma Proteiformis Essential Oil. Nat. Prod. J..

[85] Paradiso V.M., Flamminii F., Pittia P., Caponio F., Mattia C.D. (2020). Radical Scavenging Activity of Olive Oil Phenolic Antioxidants in Oil or Water Phase during the Oxidation of O/W Emulsions: An Oxidomics Approach. Antioxidants.

[86] Rodríguez G., Squeo G., Estivi L., Quezada Berru S., Buleje D., Caponio F., Brandolini A., Hidalgo A. (2021). Changes in Stability, Tocopherols, Fatty Acids and Antioxidant Capacity of Sacha Inchi (Plukenetia Volubilis) Oil during French Fries Deep-Frying. Food Chem..

